# A new species of *Hangangbathynella* (Crustacea, Bathynellacea, Parabathynellidae) from South Korea

**DOI:** 10.3897/zookeys.1046.66141

**Published:** 2021-06-21

**Authors:** Su-Jung Ji, Chi-Woo Lee, Gi-Sik Min

**Affiliations:** 1 Department of Biological Sciences, Inha University, Incheon 22212, Republic of Korea Inha University Incheon Republic of Korea

**Keywords:** Bathynellacea, COI, groundwater, *
Hangangbathynella
*, morphology

## Abstract

A new parabathynellid bathynellacean species, *Hangangbathynella mihoensis***sp. nov.**, was found in the groundwater of the Geumgang River in South Korea. This is the first report of *Hangangbathynella* from a tributary of the Geumgang River. All previously-reported species were found in the Hangang River and the origins of the two rivers are distinct from each other. The new species can be distinguished from its congeners by the two-segmented mandibular palp and the absence of epipods on thoracopod II. In this study, we provide a description of the new species and an identification table for the genus *Hangangbathynella*. In addition, we obtained partial sequences of the mitochondrial cytochrome *c* oxidase subunit I gene for DNA barcoding.

## Introduction

Bathynellacea Chappuis, 1915 is an order of groundwater crustaceans, known to have high levels of endemism owing to their limited dispersal capacity (Camacho et al. 2012). The group exclusively inhabits fresh and brackish subterranean waters and is distributed throughout all continents except Antarctica (Camacho et al. 2012). They have no eyes, simplified appendages, and a reduced number of segments in the antennule, antenna, mouthpart structures and thoracopods. As opposed to other adult malacostracans, the appendages of adult Bathynellacea are similar to those of their larvae (Schminke 1981).

The taxonomic study of Korean bathynellaceans was begun by Morimoto (1970), who described seven species through a speleological survey: two species belonging to Parabathynellidae and five belonging to Bathynellidae. To date, 30 species of the family Parabathynellidae, belonging to five genera, have been described from South Korea (Morimoto 1970; Cho et al. 2008; Park and Cho 2008, 2013, 2015a, b, 2016; Schminke 2011; Park and Eun 2012; Shin 2014): 17 species of *Allobathynella* Morimoto & Miura, 1957, two of *Eobathynella* Birstein & Ljovuschkin, 1964, four of *Nipponbathynella* Schminke, 1973, three of *Arisubathynella* Park & Eun, 2012, and four of *Hangangbathynella* Park & Cho, 2013.

As suggested by the generic name, the genus *Hangangbathynella* was first discovered and established at Hangang River in South Korea by Park and Cho (2013) and has been recorded, to date, only in the tributary of the Hangang River.

*Hangangbathynella* specimens were first collected during field surveys of groundwater habitats of the tributary of the Geumgang River in the Korean Peninsula. Based on the morphological examination of these specimens, we herein describe and illustrate them as a new species. In addition, we obtained mitochondrial cytochrome *c* oxidase subunit I (COI) gene sequence data for molecular barcoding and provide an identification table to species of the genus *Hangangbathynella*.

## Materials and methods

**Figure 1.**
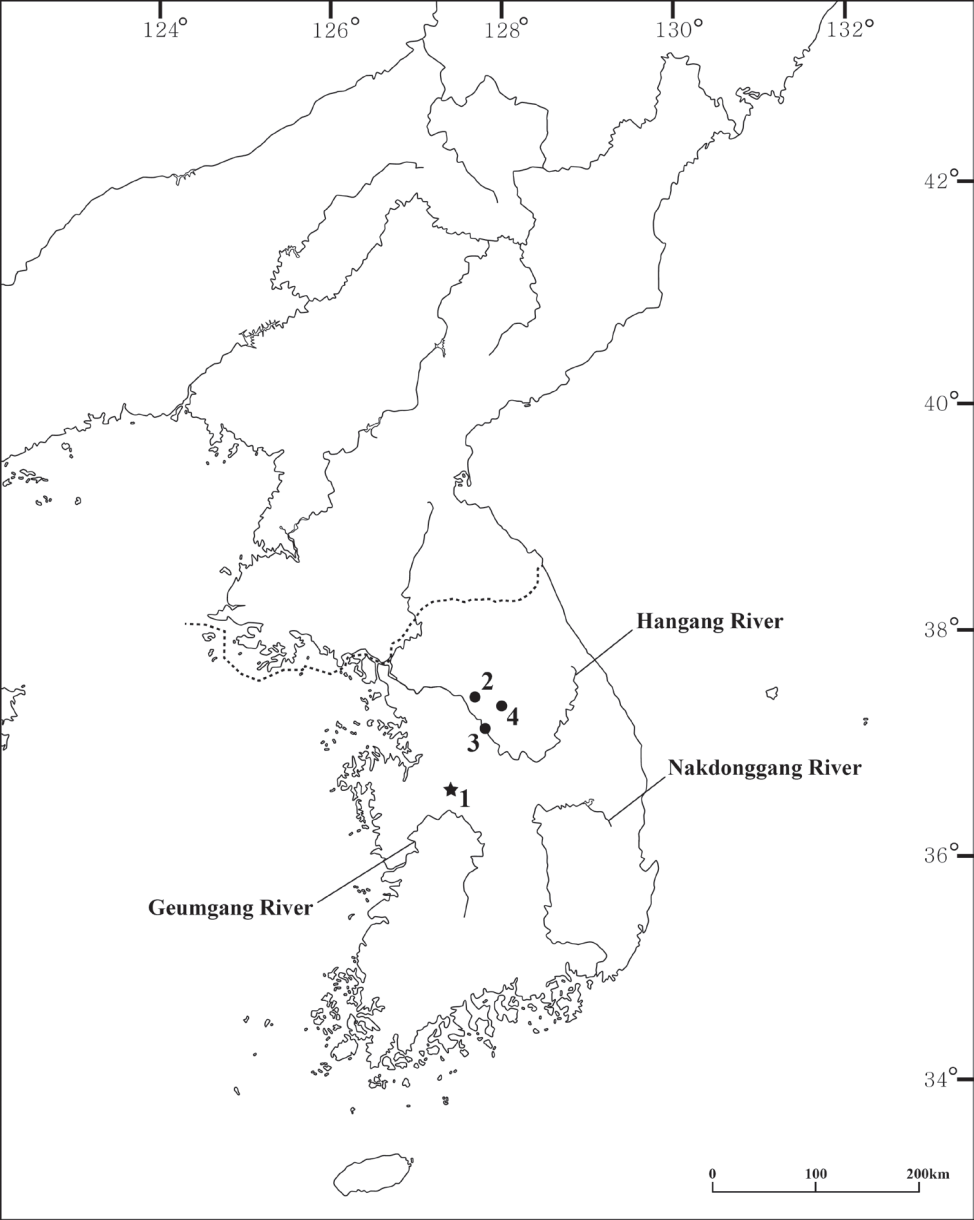
Distribution of species of the genus *Hangangbathynella* Park & Cho, 2013 **1***H. mihoensis* sp. nov. **2***H. eunokae* Park & Cho, 2013 **3***H. taechooni* Park & Cho, 2013 **4***H. karanovicae* Shin, 2014 and *H. karanovici* Shin, 2014.

*Hangangbathynella* specimens were collected from the interstitial groundwater of sandbanks near the Miho Stream (tributary of the Geumgang River) in South Korea (Fig. 1). The groundwater was pumped using a core (Lee and Park 2016) and filtered using a 50 μm fine-mesh net. The specimens were immediately fixed in 95% ethanol and dissected in glycerol under a stereomicroscope (SZX12, Olympus, Japan). Dissected appendages were mounted using Eukitt Quick-hardening mounting medium (Sigma-Aldrich, St. Louis, MO, USA) for permanent slides. Observations and drawings were performed using an optical microscope at 1000× magnification (DM2500, Leica, Germany). Specimens for scanning electron microscopy (**SEM**) were dehydrated in serial ethanol solutions, transferred into hexamethyldisilazane (Sigma-Aldrich, St. Louis, MO, USA), covered with platinum, and observed using a Hitachi SEM model S-4300SE (Hitachi, Japan). The materials were deposited in the collection of the National Institute of Biological Resources (**NIBR**), Incheon, South Korea.

Genomic DNA was extracted using the LaboPass Tissue Genomic DNA Isolation Kit Mini (Cosmo GENETECH, Seoul, South Korea) according to the manufacturer’s instructions. Partial COI sequences were obtained using the primers Bathy_F1 (5‘-ACWAAYCAYAAAGATATYGGRAC-3‘) and Bathy_R1 (5‘-CCCCCTCGAGCTTGTACAGCTCGTCCATGC-3‘). Polymerase chain reaction amplification was conducted under the following conditions: pre-denaturation at 94 °C for 2 min, followed by denaturation at 95 °C for 20 s, annealing at 48 °C for 20 s and extension at 72 °C for 1 min for 40 cycles, with a final extension at 72 °C for 5 min.

## Systematic account

**Order Bathynellacea Chappuis, 1915**

### Family Parabathynellidae Noodt, 1965

#### Genus Hangangbathynella

Taxon classificationAnimaliaBathynellaceaParabathynellidae

Park & Cho, 2013

D16EEA16-2548-5679-9564-7CA22CD35B0D

##### Diagnosis of the genus.

Body elongated and cylindrical. Antennule seven-segmented. Antenna seven-segmented. Incisor process of mandible with four teeth, a triangular proximal tooth, and molar process with more than six spines, with distal spine furcated distally. Maxilla four-segmented without medial seta on second segment. Exopod of thoracopods I–VII two-segmented. First endopodal segment of thoracopods I–VII with one tiny seta on outer distal margin. Male thoracopod VIII oval with massive protopod; epipod present in the form of gourd-shaped process reaching penial region; basis approximately half the size of protopod, without basial setae; endopod small, with two apical setae; exopod with two distal protuberances. Female thoracopod VIII as a one-segmented small process with tiny teeth and two distal setae. Pleopod absent. Uropod: protopod sympod with a row of homonomous spines; endopod with a spur distally, with two distal setae and two plumose setae on dorsal surface; exopod with a basi-ventral seta. Pleotelson with one lateral seta on each side. Anal operculum protruded. Furcal rami with four to six spines, and one long plumose seta, and one simple seta.

##### Type species.

*Hangangbathynella taechooni* Park & Cho, 2013

##### Other species.

*H. eunokae* Park & Cho, 2013, *H. karanovicae* Shin, 2014, and *H. karanovici* Shin, 2014.

#### Hangangbathynella mihoensis sp. nov.

Taxon classificationAnimaliaBathynellaceaParabathynellidae

F4E2BC4D-B638-5941-8A79-BE12B5AB2367

http://zoobank.org/66F67C7D-E5E0-4C35-B7AA-2208792C239F

##### Type locality.

Seokhwa-ri (36°38'09"N, 127°21'27"E), Cheongju-si, Chungcheongbuk-do, Korea. On a sandbank of the Miho Stream, collected by Hee-Min Yang and Su-Jung Ji (31 May 2019).

##### Type material.

***Holotype*:** male, dissected on 9 slides (NIBRIV0000879484). ***Allotype***: female, dissected on 12 slides (NIBRIV0000879485). ***Paratypes***: 9 females (NIBRIV0000879486, NIBRIV0000879487, NIBRIV0000881724, and NIBRIV0000881737–881742) and 6 males (NIBRIV0000881731–881736).

##### Diagnosis.

Antennule seven-segmented without aesthetascs on the fifth segment, and with three simple setae on inner distal margin of the sixth segment; antenna seven-segmented with setal formula 0 + 0/0 + 0/1 + 0/1 + 1/0 + 0/0 + 2/4(1); labrum with 14 teeth; mandible palp two-segmented; maxilla four-segmented with setal formula 3-3(2)-10-6; exopod of thoracopods I–VII two-segmented; thoracopods III–VII each with an epipod; inner protuberance of male thoracopod VIII with three distal spinules; female thoracopod VIII with two teeth and two distal setae; uropod sympod with 8 to 10 spines of similar size; furcal ramus with 4 to 5 spines; anal operculum protruded.

##### Description of adult male

**Figure 2.**
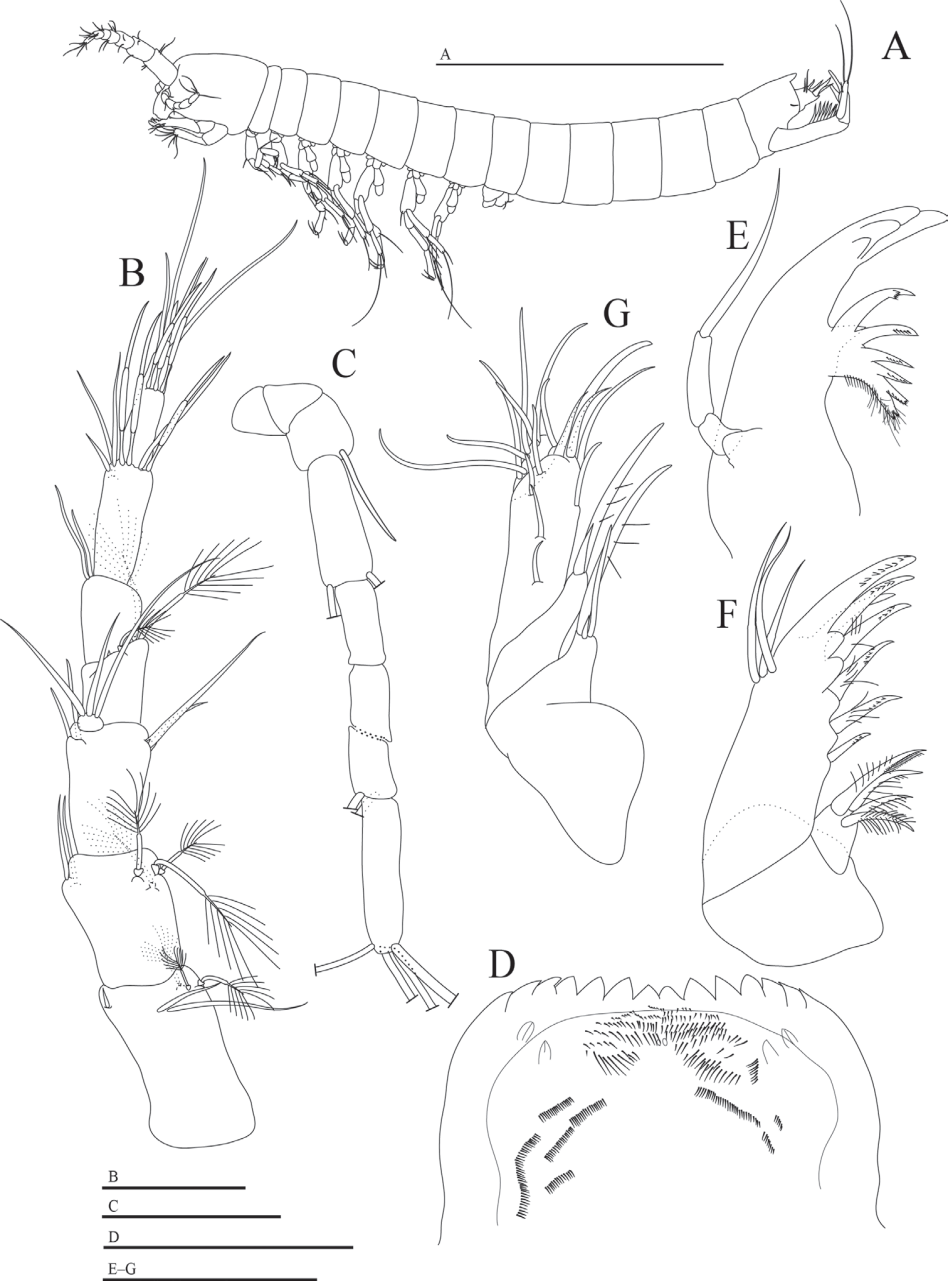
*Hangangbathynella mihoensis* sp. nov., holotype male **A** habitus (lateral) **B** antennule (dorsal) **C** antenna (ventral) **D** labrum (ventral) **E** mandible (dorsal) **F** maxillule (dorsal) **G** maxilla (dorsal). Scale bars: 0.5 mm (**A**); 0.05 mm (**B–G**).

**Figure 3.**
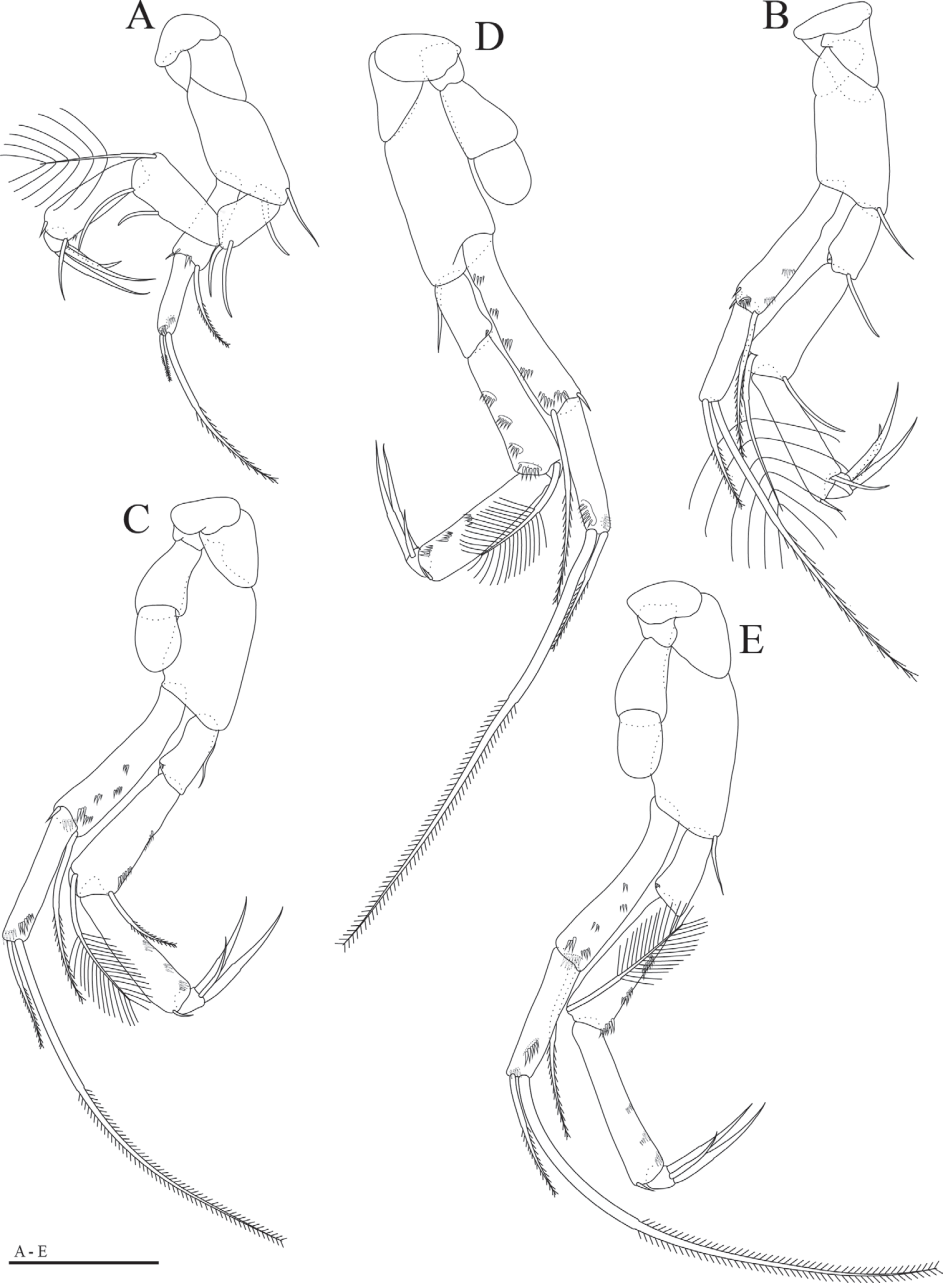
*Hangangbathynella mihoensis* sp. nov., holotype male **A–E** thoracopods I–V. Scale bar: 0.05 mm.

**Figure 4.**
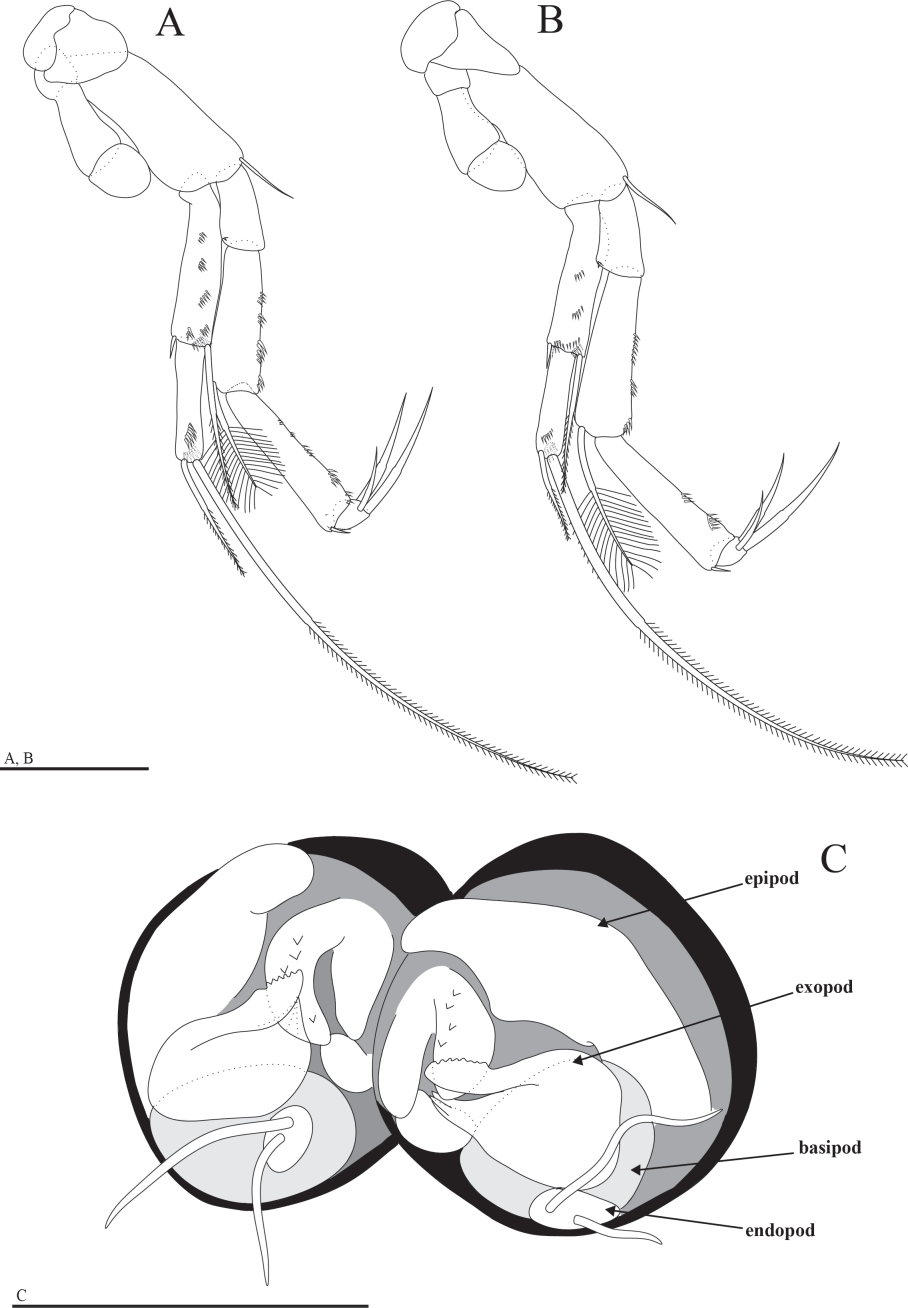
*Hangangbathynella mihoensis* sp. nov., holotype male **A** thoracopod VI (frontal) **B** thoracopod VII (frontal) **C** thoracopod VIII (ventral). Scale bars: 0.05 mm.

**Figure 5.**
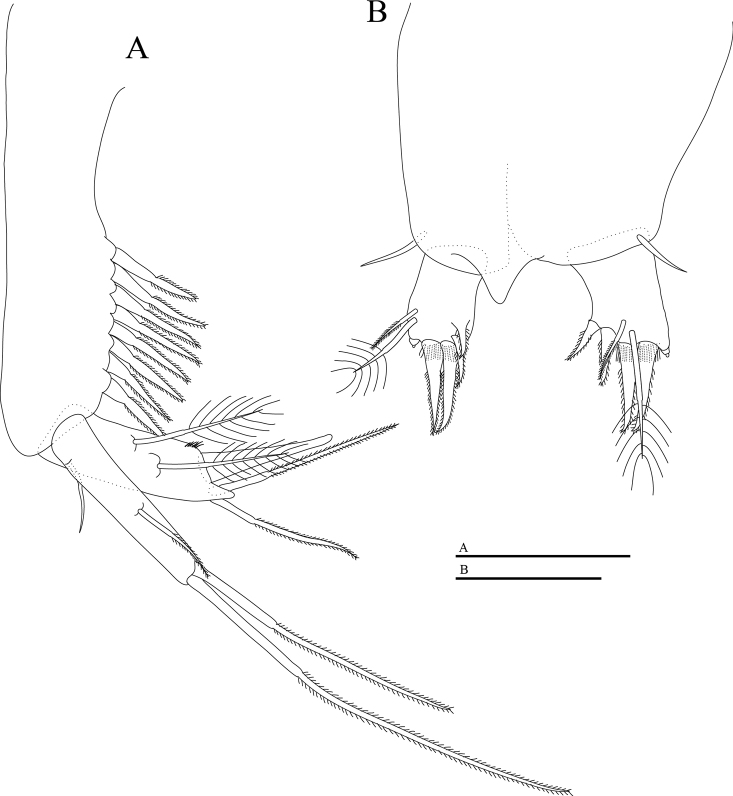
*Hangangbathynella mihoensis* sp. nov., holotype male **A** pleotelson and furcal rami (dorsal) **B** uropod (lateral). Scale bars: 0.05 mm.

**(Figs 2–5).** Body length 1.13 mm (Fig. 2A), approximately 10 times as long as wide. Head as long as anterior three thoracic segments combined.

Antennule (Fig. 2B) seven-segmented, first segment with one small seta on inner distal margin, two simple dorsal setae of different sizes, three plumose setae on outer margin; second segment with two simple setae on inner distal margin, one group of four plumose setae on outer margin; third segment with one simple dorsal and one ventral seta on inner distal margin, and with two simple setae of different sizes and one tiny seta on outer margin; inner flagellum of third segment with three simple setae; fourth segment with one stub seta and one plumose seta on dorsal margin, two stub setae and two plumose setae on outer distal apophysis, which is slightly protruded; fifth segment with one dorsal simple seta, distally with two setae on inner distal margin; sixth segment with three simple setae on inner distal margin, two aesthetascs and one simple seta dorsally, one aesthetasc on outer margin; seventh segment with three subterminal aesthetascs and four distal simple setae.

Antenna (Fig. 2C) seven-segmented; as long as antennular segments 1–6 combined; setal formula 0 + 0/0 + 0/1 + 0/1 + 1/0 + 0/0 + 2/4.

Labrum (Fig. 2D) flat with eight median teeth of similar size, two central teeth slightly smaller than the rest, flanked by three teeth on each side; inner surface with two pairs of nipple-like lateral protrusions and with ctenidia and two tiny projections in middle region.

Mandible (Fig. 2E) with incisor process of four teeth; tooth of ventral edge triangular; spine row consisting of seven spines; palp two-segmented with one apical seta not exceeding incisor process in length.

Maxillule (Fig. 2F) two-segmented, proximal segment with four setae on distal margin; distal segment with two terminal dentated spines; five dentated spines on inner edge, and three simple setae on outer distal margin.

Maxilla (Fig. 2G) four-segmented, setal formula 3-3(2)-10-6.

Thoracopods I–VII (Figs 3A–E, 4A, B) slightly increasing in size up to thoracopod IV, thoracopods IV–VII similar in size; thoracopods III–VII each bearing one epipod on protopod; basis of thoracopod I with two setae, that of thoracopods II–VII with one seta; exopod of thoracopods I–VII two-segmented; endopods of thoracopods I–VII four-segmented, setal formulae:

**Table:** 

Thoracopod I	2 + 1/2 + 1/1 + 1/3(1)
Thoracopods II	1 + 1/1 + 1/1 + 1/3(1)
Thoracopods III, IV	0 + 1/1 + 1/0 + 1/3(1)
Thoracopods V–VII	0 + 1/0 + 1/0 + 1/3(1)

**Figure 6.**
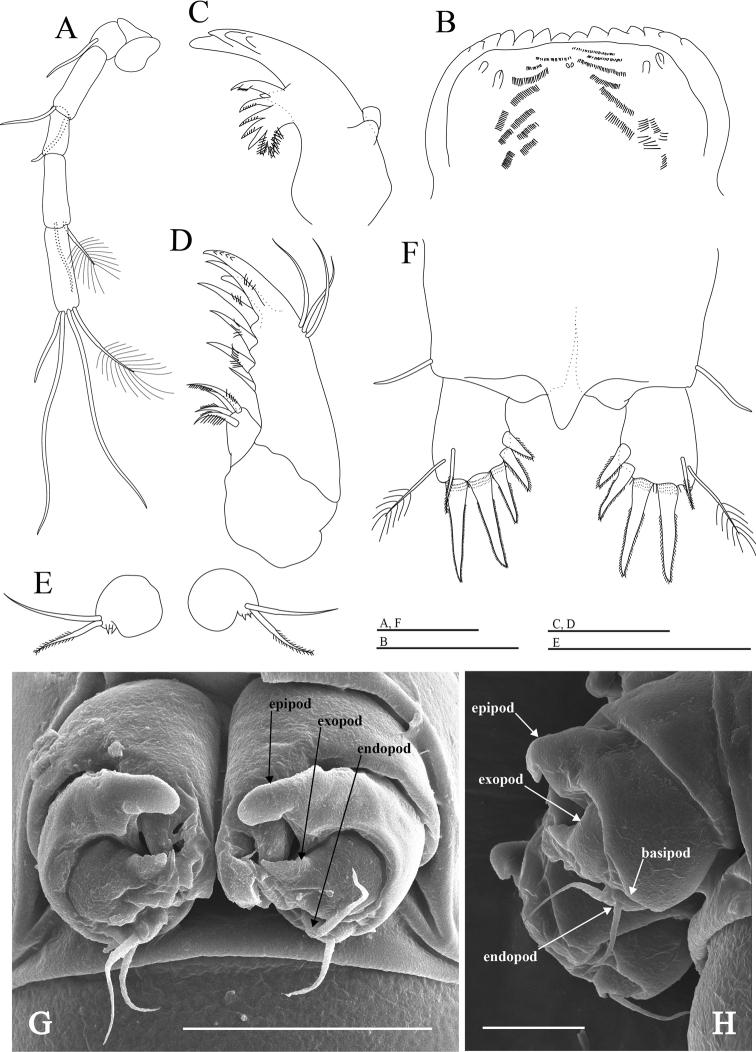
*Hangangbathynella mihoensis* sp. nov. **A–D, F** paratype female (NIBRIV0000881724) **E** allotype female **G, H** paratype male (NIBRIV0000881731) **A** antenna (ventral) **B** labrum (ventral) **C** mandible (dorsal) **D** maxillule (dorsal) **E** thoracopod VIII (ventral) **F** pleotelson and furcal rami (dorsal) **G** thoracopod VIII (ventral) **H** thoracopod VIII (lateral). Scale bars: 0.05 mm (**A–G**); 0.02 mm (**H**).

Thoracopod VIII (Figs 4C, 6G, H) nearly rectangular in lateral view; main axis tilting backwards; protopod massive with prominent penial region displaying distal opening encircled by frontal, median lobes and dentated inner lobe with four dentils; epipod large, gourd-shaped, distal part hardly covering penial region; basis as large as one half of protopod, without setae; exopod round, one half of basis with two distal protuberances; outer protuberance serrated, inner one with three distal spinules; endopod small, with two distal setae.

First pleopod absent (Fig. 2A).

Uropod (Fig. 5A) with load-shaped sympod bearing eight spines of similar size on inner margin; endopod 28% as long as sympod length, with one large distal spur, two barbed setae and two plumose setae on dorsal surface; exopod longer than endopod, 50% as long as protopod, with one outer seta, two terminal setae and one inner medial seta; inner setae strong, longer and thicker than outer terminal seta.

Pleotelson (Fig. 5B) with one lateral seta on each side.

Anal operculum (Fig. 5B) protruded.

Furcal rami (Fig. 5B) slightly longer than wide, with four spines and two setae, longer one plumose and shorter one barbed.

##### Description of adult female.

The female differs from the male in thoracopod VIII. Thoracopod VIII (Fig. 6E) as a gourd-shaped protuberance with two distal setae and two tiny spines.

Antenna (Fig. 6A) seven-segmented, two proximal segments without setae; third segment with one simple seta on inner edge; fourth segment with one simple seta on inner edge and one simple seta on outer distal margin; fifth segment without setae; sixth segment with one simple and one plumose seta on outer distal margin; distal segment with three simple setae and one plumose seta. Labrum (Fig. 6B) with 14 teeth. Mandible (Fig. 6C) with incisor process of four teeth, tooth of ventral edge triangular, spine row consisting of seven spines. Maxillule (Fig. 6D) two-segmented.

##### Variation.

Body length 0.91–1.14 mm in female (six individuals, NIBRIV0000881737–881742), 1.04–1.13 mm in male (five individuals, NIBRIV0000881732–881736). Uropod protopod with eight to nine, rarely 10 spines. Furcal rami mostly with four spines but five on one side in NIBRIV0000881724 (Fig. 6F).

##### Etymology.

The species name is derived from the stream (Miho, a tributary of the Geumgang River) in which the species was collected.

##### Molecular analysis.

A total of 606 base pairs of COI sequences (GenBank accession numbers: MW429327, MW429328, and MW624440) were obtained from three individuals of *Hangangbathynella mihoensis* sp. nov. (NIBRIV0000879486, NIBRIV0000879487, and NIBRIV0000881724). The intra-specific distance of the COI sequences ranged from 0.5% to 1.0%. Although the phylogenetic relationships of *Hangangbathynella* could not be confirmed owing to the lack of molecular data on Korean bathynellaceans, this result suggests that COI will be useful in distinguishing Korean bathynellacean species in the future.

## Discussion

The new species belongs to the genus *Hangangbathynella*, and species of this genus have been recorded only in the Hangang River to date (Park and Cho 2013). However, the new species was first found in the Miho Stream, which is a tributary of the Geumgang River (Fig. 1).

*Hangangbathynella mihoensis* sp. nov. most closely resembles *H. taechooni* in the following characteristics: 1) the antennule’s 6^th^ segment bears three simple setae on inner distal margin, 2) the antenna’s 4^th^ segment bears two setae, and 3) the antenna’s 6^th^ segment bears one simple and one plumose seta. However, the new species differs from *H. taechooni* by the following features (features of *H. taechooni* in parentheses): 1) the labrum has 14 (12) teeth, 2) the mandibular palp is two (one) segmented, and 3) thoracopod I has two (one) basipod setae.

**Table 1.** Morphological differences among the five species of *Hangangbathynella*. Abbreviation: th. = thoracopod.

		*H. taechooni*	*H. eunokae*	*H. karanovicae*	*H. karanovici*	*H. mihoensis* sp. nov.
Labrum	no. teeth	2+8+2	2+8+2	2+10+2	2+8+2	3+8+3
Mandible	palp segment	1	1	1	1	2
	no. of spines in spine row	7	8	7	7	7
Thoracopods	epipod in th.2	present	present	present	present	absent
	no. basipod setae on th.1	1	2	2	2	2
Male th.8	no. protuberances on exopod	2	1	2	2	2
Female th.8	no. spinules	3	3	8	4	2
Uropod	no. spines of protopod	10–11	9	10	11	8–9
Furcal rami	no. of spines	5	5	5	5–6	4–5

*Hangangbathynella mihoensis* sp. nov. is morphologically similar to *H. karanovicae* in the following characteristics: 1) the labrum has 14 teeth, 2) thoracopod I has two basipod setae, and 3) the exopod of male thoracopod VIII has two protuberances. However, *H. mihoensis* sp. nov. differs from *H. karanovicae* by the following features (features of *H. karanovicae* in parentheses): 1) the maxilla’s 1^st^ segment bears three (two) setae, 2) the epipod in thoracopod II is absent (present), and 3) female thoracopod VIII has two (seven) spinules.

*Hangangbathynella eunokae* and *H. karanovici* also share the following characteristics with the new species: 1) the labrum has eight median teeth and 2) thoracopod I has two basipod setae. However, *H. mihoensis* differs from these two species by the following features: from *H. eunokae* (features of *H. eunokae* in parentheses), 1) the antennule’s 6^th^ segment bears three (two) simple setae on the inner distal margin, 2) the antenna’s 6^th^ segment bears one simple and one plumose seta (two simple setae), and 3) the exopod of male thoracopod VIII has two (one) protuberances; from *H. karanovici* (features of *H. karanovici* in parentheses), 1) the antennule’s 6^th^ segment bears three (two) setae on the inner distal margin, 2) the antenna’s 4^th^ segment bears two (one) setae, and 3) female thoracopod VIII has two (four) spinules.

## Supplementary Material

XML Treatment for Hangangbathynella

XML Treatment for Hangangbathynella mihoensis
